# Acute circulatory and femoral hemodynamic responses induced by standing core exercise at different rotational cadence: a crossover study

**DOI:** 10.1186/s13102-022-00589-w

**Published:** 2022-11-17

**Authors:** Hsin-Fu Lin, Chun-Chung Chou, Hsiao-Han Chao, Soun-Cheng Wang, Chen-Huan Chen

**Affiliations:** 1 Department of Athletics, National Taiwan University, No. 1., Sec 4, Roosevelt Rd., Taipei, 10617 Taiwan; 2 Office of Physical Education, National Taipei University of Technology, Taipei, Taiwan; 3 Department of Athletic Sports, National Chung Cheng University, Chiayi, Taiwan; 4 Department of Medicine, National Yang Ming Chiao Tung University College of Medicine, Taipei, Taiwan

**Keywords:** Strength endurance exercise, Trunk muscle, Muscle endurance

## Abstract

**Background:**

Core exercise is often adopted as an adjunct in maintaining musculoskeletal health in rehabilitation; we previously showed that standing core rotational exercise improves femoral blood flow after training. This study aimed to investigate the effects of different rotational cadences on circulatory and hemodynamic responses after acute standing core exercise.

**Methods:**

Sixteen healthy male adults (22 ± 1 yrs) were randomly assigned to participate in two 30-min standing core exercises of fast (75 rpm, FC) and slow cadence (20 rpm, SC) sessions after completing an acute bout of seated knee extension exercise session (KE) (80% of 1 repetition maximum × 12 repetitions × 3 sets). Impedance cardiography-derived circulatory responses and femoral hemodynamics by ultrasound imaging were measured pre- and 30, and 60 min post-exercise.

**Results:**

KE acutely increased post-exercise cardiac output at 30 min (*p* = 0.008) and heart rate at 30 min (*p* = 0.04) and 60 min (*p* = 0.01), yet brachial blood pressure did not change. Systemic vascular resistance was significantly lower after FC and KE at 30 min (*p* = 0.008) and 60 (*p* = 0.04) min, respectively, compared with the baseline. In addition, KE acutely decreased post-exercise arterial stiffness (*p* = 0.05) at 30 min, increased femoral conductance (*p* = 0.03, *p* < 0.001), and blood flow (*p* = 0.009, *p* < 0.001) at 30 and 60 min. No significant changes were observed in absolute femoral blood flow after FC and SC, except that FC significantly increased relative femoral blood flow (*p* = 0.007) and conductance (*p* = 0.005). Post-exercise femoral diameter significantly increased in KE at 30 (*p* = 0.03) and 60 min (*p* = 0.01), but not in core exercise.

**Conclusion:**

Our results suggest that standing core exercise elicits circulatory and hemodynamic changes only when the rotational cadence is set at a faster cadence, which provides preliminary scientific evidence for its use in exercise programs.

## Introduction

Habitual exercise is believed to increase blood flow via the increase in shear stress and endothelial function, thereby preserving vascular health [1, 2] and improving oxygen delivery and utilization for better exercise performance [3]. On the contrary, reduced physical activity as the result of injury, for example, attenuates the cardiovascular benefits gained by exercise training [4, 5]. Therefore, staying active post-surgery or rehabilitation is critical in mitigating the deterioration of vascular function and boosting energy expenditure.

**Fig. 1:**
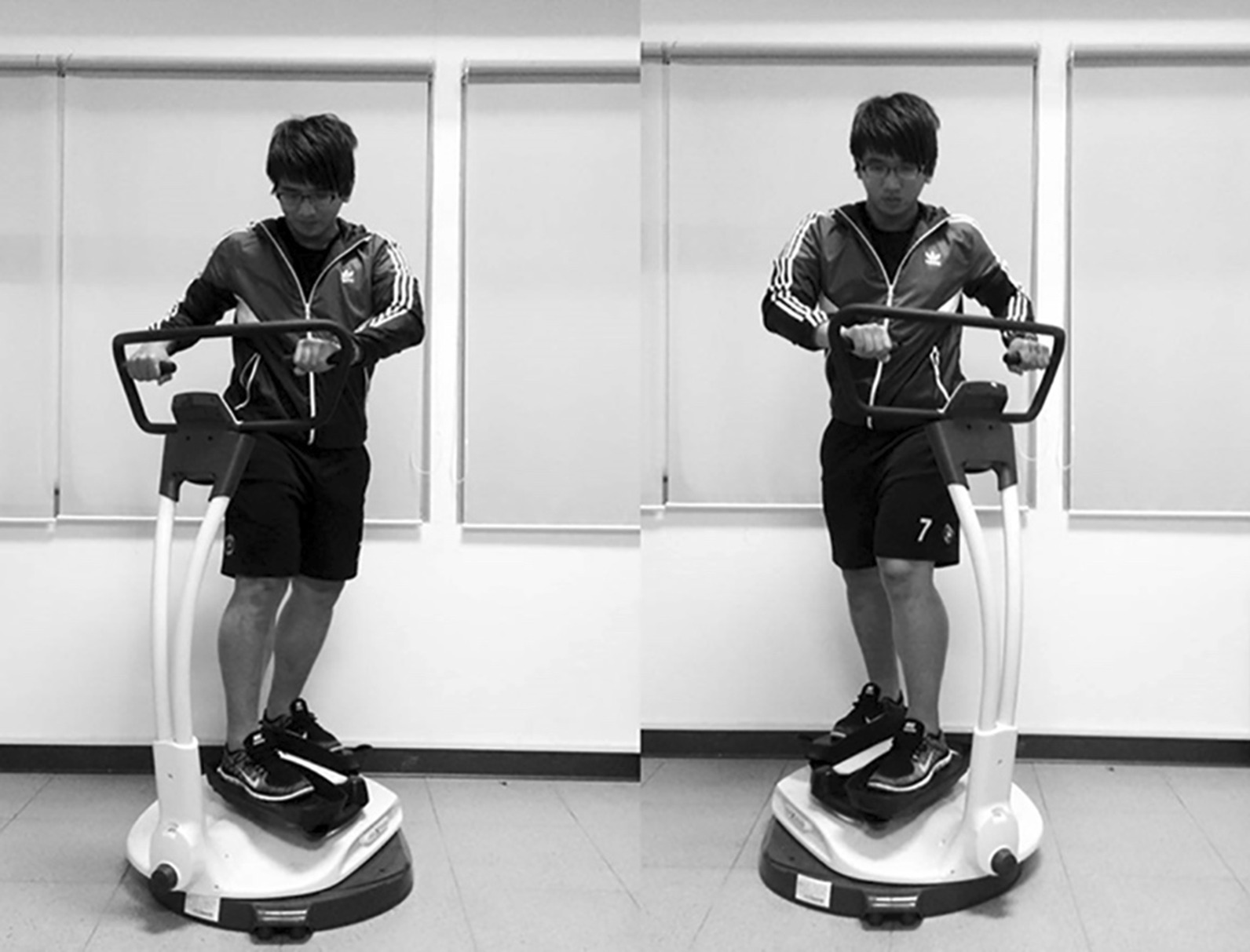
Scheme of core rotational exercise (the subject has permitted the use of this figure)

Core muscles provide proximal stability during limb movements and lumbopelvic stability [6]. Core exercise training has been demonstrated to benefit young athletes [7], older adults [8, 9], and stroke patients [10] by improving trunk function, stability, and mobility, which are critical to reconstruct physical functions. Traditionally core exercises are performed on the floor, which is not accessible for injured individuals. We recently adopted a standing core rotational exercise device that provided low resistance from the platform against arm-assisted trunk rotation (Fig. 1). We demonstrated that femoral blood flow increases after short-term training [11]. Such exercise has some essential features. First, its trunk rotational movements uniquely mimic a particular core function pattern needed during daily tasks and sports activities [6]. Moreover, a standing position could elicit a more significant core neuromuscular activation during exercise [12]. Its smooth neutral movement characterizes this exercise mode by coordinating the trunk and pelvis in the axial rotation critical for walking [13]. However, the extent to which the metabolic and cardiovascular responses elicited by acute core muscle-biased exercise is not fully understood in the current literature, which limits its clinical use when designing an exercise program.

In this study, we aimed to investigate the metabolic and cardiovascular responses of standing core exercise in young adults who have higher responsiveness to exercise stimulus in increasing blood flow than the elderly [14]. We tested the hypothesis that acute standing core exercise at a faster cadence could elicit higher circulatory and hemodynamic changes following exercise than at a slower cadence.

## Methods

### Experimental design

After screening, all eligible participants underwent a familiarization that included a blood pressure measurement, a fasting blood draw, familiarity with the exercise device, and a one-repetition maximum (1RM) strength test on knee extension exercise [15]. Muscle mass was also determined noninvasively using dual-energy X-ray absorptiometry (DEXA) (Lunar DPX, GE Fairfield, CT). Using a standard protocol [15], participants underwent a maximal treadmill exercise test during the first visit to obtain individual peak oxygen consumption (VO2 peak) by using a metabolic cart (MetaLyzer 3B, Cortex, Germany). In the second visit,  they also performed two core rotational exercise trials with the metabolic cart to characterize physiological responses at different cadences. To effectively address our aim, we incorporate seated knee extension exercise (KE) as the active control, which has been demonstrated to increase blood flow [16] and is commonly used for rehabilitation. After the screening visit, subjects performed a single bout of KE exercise followed by either fast core (FC) or slow core (SC) exercise in a randomized order.

Participants performed three sets of knee extension muscle contractions for KE, producing 90–170° arc movement, using both legs simultaneously. The exercise protocol consisted of 12 repetitions in the first two sets, followed by repetitions to fatigue with verbal encouragement in the third set. Exercise intensity was set at 80%1RM for all sets. Participants were allowed to rest for 2 min between the sets. In the core exercise sessions, participants used a core exercise machine equipped with adjustable resistance (Core X. Fitcrew Inc., Taiwan), as shown in our previous work [11]. Participants were instructed to rotate their trunk within a set range of rotation (at least 140°). Handgrip assistance in the same position was allowed to guard against the machine’s spring resistance (13.5 kg). Hence, during exercise, participants were required to work against this spring resistance to achieve the required range of motion for 30 min. We provided a metronome cadence for participants during exercise; a cadence of 75 rpm for FC and 20 rpm for SC was set as different exercise intensities. To our knowledge, no study had determined the effects of cadence on physiological responses in this exercise mode; we chose the cadence based on our participants’ comfort from our pilot work and the previous study [11]. All participants were able to complete all exercise protocols with verbal encouragement.

### Participants

Sixteen healthy, recreational young active male adults were recruited to participate in this study after screening. Exclusion criteria included: [1] obesity (BMI > 30 kg/m^2^) [2], cigarette smoking within the past six months [3], history of diabetes (fasting blood glucose > 126 mg/dL) and heart disease [5], injury that may prevent him or her from completing the exercise; and [6] use of over-the-counter supplements or vitamins. None of the participants exercised (resistance or endurance training) more than three times a week, as determined by a physical activity questionnaire. Recruited participants had no history of hypertension (< 140/90 mmHg) or kidney dysfunction. All participants gave their written informed consent; all procedures were performed in accordance with relevant guidelines and approved by the Institutional Review Board of the school hospital (ID:201112135RIC).

### Procedures

Measurements were conducted before, 30, and 60 min after exercise. Participants were asked to rest in a supine position during testing sessions. All tests were performed at the same time of day for each participant. Exercise bouts were at least 24 h apart. Participants were asked to maintain their regular diet and avoid strenuous exercise between testing sessions. They were instructed to arrive at the laboratory after a 12-h overnight fast.

### Measurements

#### Cardiovascular response

Impedance cardiography (ICG) was performed using a non-invasive cardiac output module (SS31LA, Biopac, Goleta CA) and an electrocardiogram (ECG) sensor (SS2L) connected to a physiological signaling processing system (MP36, Biopac, Goleta CA) to determine stroke volume (SV), heart rate (HR) and cardiac output (CO) by following the manufacturer’s instructions and the guidelines [17]. Mean arterial blood pressure (MAP) was determined by the automatic vascular testing device (MAP = 1/3SBP + 2/3DBP). Systemic vascular resistance (SVR) was also calculated by the equation: SVR (dyne/cm^2^) = 80 × MAP/CO [17]. The day-to-day coefficients of variation for BP and CO in our laboratory were less than 3% and 10%, respectively.

#### Vascular function

The participants were instructed to rest quietly in the supine position for at least 10 min before measurement. Brachial blood pressure, systemic arterial stiffness, and brachial-ankle pulse wave velocity (baPWV) were obtained using an automated vascular testing device (VP-1000 plus, Omron Healthcare). All measurements were duplicated, and average values were used for subsequent analyses.

#### Leg hemodynamics

Longitudinal images of the common carotid artery 1-2 cm proximal to the bifurcation were obtained noninvasively in the supine position using an ultrasound machine (Sonosite Ultrasound System, Bothell, WA). Vessel diameter was determined at a perpendicular angle along the scanned area axis. Blood velocity was assessed with the transducer appropriately positioned to maintain an insonation angle at 60° or less. Images were analyzed using image analysis software (ImageJ, NIH, Bethesda, MD) that included at least ten consecutive cycles. Femoral blood flow was calculated as mean blood velocity × π × (femoral diastolic diameter/2) ^2^ × 60, ml/min [18]. Femoral conductance was calculated as femoral blood flow/mean arterial pressure. An independent investigator also performed all ultrasound imaging and analyses, and our laboratory’s day-to-day coefficients of variation for femoral artery diameter and velocity are 1.62 and 1%, respectively.

#### Statistical analyses

Statistical analyses were performed using Graph Pad Prism 8.0 (La Jolla, CA). All data are reported as mean ± SEM. Two-way repeated-measures ANOVA with a Bonferroni post hoc analysis was used to determine the effects of exercise and time on measured parameters. Significance was set *a priori* at *p* < 0.05.

## Results

Physiological and metabolic responses for FC and SC and participants’ characteristics are presented in Tables 1 and 2. FC and SC’s intensity was 52%, 47% of maximum heart rate (HRmax), and 43% and 28% of individual VO_2_ peak, respectively. As shown in Table 3, brachial BP was not significantly affected by any of the exercise bouts. BaPWV was significantly reduced 30 min post-exercise in KE (*p* = 0.05), whereas no changes were observed in FC and SC sessions.

**Table 1:** Selected subject characteristics

	Mean ± SEM (N = 16)
Age, yrs	22 ± 1
Height, cm	171 ± 2
Weight, kg	65 ± 3
BMI, kg (m^2^)^−1^	22 ± 1
Leg lean muscle mass, kg	17 ± 1
Percent body fat, %	20 ± 1
Total cholesterol, mg dl^− 1^	167 ± 5
HDL cholesterol, mg dl^− 1^	53 ± 2
LDL cholesterol, mg dl^− 1^	98 ± 4
CRP, mg dl^− 1^	0.04 ± 0.01
Fasting glucose, mg dl^− 1^	85 ± 3
Brachial SBP, mmHg	113 ± 2
Brachial DBP, mmHg	62 ± 2
Treadmill VO_2_ peak, ml/kg/min	42.7 ± 1.1

*HDL* High-intensity lipoprotein, *LDL* Low-intensity lipoprotein, *CRP* C-reactive protein, *SBP* Systolic blood pressure, *DBP* Diastolic blood pressure, *VO*_2_ *peak* Peak oxygen uptake

**Table 2:** Physiological characteristics of core exercise

	Fast core (75 rpm)	Slow core (20 rpm)
VO_2_, ml/kg/min	18.6 ± 0.7	12.0 ± 0.7
HR, bpm	103 ± 4	93 ± 3
%VO_2_ peak	43 ± 2	28 ± 2
%HRmax	52 ± 2	47 ± 1
RER	0.86 ± 0.04	0.78 ± 0.02

HR, Heart rate; %VO_2_ peak, Percentage of peak oxygen consumption (steady oxygen consumption obtained from core exercise/treadmill VO_2_ peak); %HRmax, Percentage of maximal heart rate (steady heart rate obtained from core exercise/treadmill HRmax), RER, Rate of exchange ratio

**Table 3:** Circulatory and vascular responses pre- and post-exercise

	Baseline	30 min post	60 min post
Brachial SBP, mmHg			
KE	114 ± 1	115 ± 2	115 ± 3
FC	115 ± 2	113 ± 2	113 ± 2
SC	114 ± 2	115 ± 3	114 ± 2
Brachial DBP, mmHg			
KE	62 ± 2	62 ± 2	61 ± 1
FC	64 ± 2	62 ± 2	62 ± 2
SC	63 ± 2	63 ± 2	62 ± 2
Heart rate, bpm			
KE	60 ± 2	68 ± 2*	64 ± 2*
FC	63 ± 3	64 ± 2^✝^	62 ± 2
SC	63 ± 3	64 ± 2^✝^	60 ± 3✝
Stroke volume, ml			
KE	59 ± 5	63 ± 4	65 ± 5
FC	64 ± 6	65 ± 5	62 ± 5
SC	66 ± 6	66 ± 5	66 ± 4
Cardiac output, ml/min			
KE	3650 ± 252	4242 ± 252*	4080 ± 261
FC	3664 ± 308	4076 ± 288	3806 ± 284
SC	4068 ± 308	4189 ± 271	3812 ± 214
MAP, mmHg			
KE	82 ± 1	83 ± 1	84 ± 2
FC	84 ± 2	83 ± 2	83 ± 2
SC	84 ± 2	83 ± 2	82 ± 2
SVR, dyne/cm^2^			
KE	1.74 ± 0.12	1.61 ± 0.13	1.51 ± 0.12*^‡^
FC	1.94 ± 0.16	1.63 ± 0.12*	1.74 ± 0.12
SC	1.74 ± 0.15	1.63 ± 0.10	1.70 ± 0.09
baPWV, cm s^− 1^			
KE	1108 ± 46	1069 ± 55*	1073 ± 52
FC	1094 ± 43	1098 ± 58	1092 ± 55
SC	1093 ± 53	1091 ± 52	1092 ± 50

Values, mean ± SEM; KE, Seated knee extension; FC, Fast core; SC, Slow core; SBP, Systolic blood pressure, DBP, Diastolic blood pressure; MAP, Mean arterial pressure, SVR, Systemic vascular resistance; baPWV, Brachia-ankle pulse wave velocity

*Versus baseline

^✝^Versus KE

^‡^Versus FC. *p* < 0.05

In terms of circulatory changes (Table 3), HR significantly elevated 30 (*p* = 0.04) and 60 min (*p* = 0.01) compared with the baseline after KE. There was no significant change in HR after FC and SC. Regardless of the exercise mode, no significant differences were shown in SV and MAP before and after exercise. CO was significantly elevated at 30 min after KE (*p* = 0.008), whereas no significant changes were observed in the core exercise. SVR significantly reduced at 60 mins post-exercise in KE (*p* = 0.04) and at 30 min after the FC trial (*p* = 0.008). Overall there was no interaction (exercise’ time) and group difference (exercise mode) in measured circulatory variables at different time points.

**Fig. 2:**
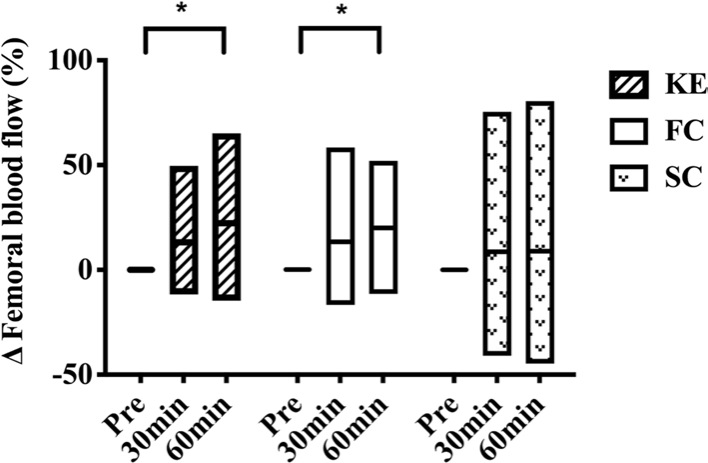
Relative change in blood flow from baseline in different exercise conditions

Common femoral blood flow consistently increased after KE and FC, but not SC. It significantly increased at 30 and 60 min after exercise in the KE trial (*p* = 0.009, *p* < 0.001), but it did not change substantially in the FC trial, despite a trend (*p* = 0.10) at 60 min. The relative change in blood flow from baseline also significantly increased at 60 min after the KE (*p* = 0.002) and the FC trial (*p* = 0.007) (Fig. 2). No group difference was found between KE and FC at different time points. Femoral conductance increased 30 and 60 min post KE exercise (*p* = 0.03, *p* < 0.001) and 60 min after FC (*p* = 0.005), whereas SC did not result in significant changes. The femoral diameter was increased 30 and 60 min after the KE trial (*p* = 0.03, *p* = 0.01), whereas no significant changes were observed in the core exercise (Table 4). There were also no time and group differences in the femoral shear rate and velocity after exercise.

**Table 4:** Femoral hemodynamic changes pre- and post-exercise

	Baseline	30 min post	60 min post
Femoral blood flow, ml min^− 1^			
KE	225 ± 15	258 ± 27*	270 ± 19*
FC	237 ± 27	252 ± 28	267 ± 29
SC	225 ± 24	221 ± 24	221 ± 27
Femoral conductance, ml/min/mmHg			
KE	2.9 ± 0.2	3.4 ± 0.3*^†^	3.6 ± 0.3*^†^
FC	3.0 ± 0.4	3.4 ± 0.4^†^	3.6 ± 0.5*^†^
SC	2.8 ± 0.3	2.9 ± 0.3	3.0 ± 0.4
Femoral shear rate, 1/s			
KE	113 ± 8	113 ± 8	117 ± 10
FC	106 ± 9	114 ± 8	108 ± 8
SC	118 ± 13	115 ± 9	117 ± 12
Femoral diameter, cm			
KE	0.88 ± 0.03	0.92 ± 0.03*	0.92 ± 0.03*
FC	0.92 ± 0.03	0.92 ± 0.03	0.93 ± 0.03
SC	0.88 ± 0.03	0.88 ± 003	0.88 ± 0.03
Femoral velocity, cm/s			
KE	24.2 ± 1.4	25.5 ± 1.7	26.2 ± 1.7
FC	23.8 ± 1.8	25.7 ± 1.5	24.8 ± 1.5
SC	24.6 ± 1.6	24.9 ± 1.7	25.1 ± 2.3

Values, Mean ± SEM. SBP, Systolic blood pressure; DBP, Diastolic blood pressure; baPWV, Brachia-ankle pulse wave velocity, KE, Knee extension; FC, Fast core; SC, Slow core

*Versus baseline

^†^Versus SC. *p* < 0.05

## Discussion

The present study aimed to determine the circulatory responses of standing core exercise and its leg hemodynamic changes following an acute bout of exercise. We first characterized the metabolic demand and found that this modality could elicit a mild response in oxygen uptake (28–43%VO_2_ peak) and heart rate (~ 50%HRmax) during exercise. To our knowledge, current literature characterizing the physiological demand of core exercise is sparse. Compared to the other similar home-based core muscle exercise that mimics horse-riding, standing core exercise elicited more significant oxygen uptake responses (~ 800ml/min vs. ~ 400ml/min) during exercise [19]. Such difference could be attributed to the extent to which muscles are recruited during exercise. Indeed, the horse-riding core exercise is non-weight bearing (sitting on the device), whereas our exercise requires subjects to stand on the device with their hands grasping the bar. Previous evidence showed that resistance exercise with a standing posture activated more core muscles than sitting [12]. Moreover, in the present study, participants were instructed to perform trunk rotations with upper arm movements against the platform’s resistance and to recruit more muscle mass than horse riding, thereby increasing metabolic demands. Nevertheless, standing core rotational exercise could be classified as a low-to-moderate intensity exercise according to the exercise guidelines [15].

It may not be surprising that KE, as the active control, results in pronounced increases in cardiac output, femoral blood flow, and conductance and a reduction in systemic vascular resistance and arterial stiffness. Such findings agree with previous studies demonstrating that KE acutely increases limb blood flow and conductance[16]. We found blood flow remained elevated in the large artery up to 60 min post-exercise, which was consistent with previous studies [20, 21]. On the contrary, core exercise did not increase post-exercise cardiac output and absolute femoral blood flow, although the significance is close to the critical value in FC. However, the relative change of femoral blood flow in FC increased following exercise (shown in Fig. 2), accompanied by continuous femoral conductance increase. Of note, our recent study has demonstrated that femoral blood flow increased after eight weeks of FC training in the older population[11], indicating repetitive episode of the core exercise was required for a long-term improvement in blood flow.

As hypothesized, FC elicits more significant circulatory and hemodynamic changes than SC. The more frequent and lower force contractions introduce higher blood flow than longer and higher force contractions [22], suggesting that exercise cadence is critical in determining blood flow when using this exercise modality. We cannot exclude the possibility that blood flow may be substantially distributed to the upper body as the exercise was performed with arm movements. Of note, we also found that some participants might have performed torso isometric contractions to maintain body posture while waiting for the next metronomic beat during the SC due to its slow cadence (20 rpm), contributing to a greater blood flow toward the torso. The isometric contractions occurring during SC may also explain our finding that %HRmax of FC was only 5% higher than that of SC in this study.

Limb blood flow is tightly coupled with the metabolic demand of contractions [22, 23]. The exercise-induced blood flow and shear stress acting on the vessel wall leads to vasodilator substance releases, such as nitric oxide, acetylcholine, endothelium-dependent dilators, and following vasodilation [23]. The blood flow from the heart and shear rate decreases with the cessation of exercise. Therefore, we believe that the increase in post-exercise femoral diameter and reduced arterial stiffness induced by KE may be associated with the residual circulating endothelium-derived vasodilator substances from active and non-active muscles [23], as well as the augmented shear-mediated sensitivity on endothelium after exercise [1]. It is also possible that blood flow and diameter increase via the so-called “ascending vasodilation” mechanism by which distal exercising muscles alter vascular smooth muscle tone [24]. On the other hand, both FC and SC do not affect vessel diameter, velocity, and arterial stiffness, consistent with our previous training study [11]. The tendency of blood flow increase is more likely attributed to the rise of post-exercise metabolic demand since resting muscle remains mechanically active [25]; higher intensity was shown to elicit a greater excess post-exercise oxygen consumption after acute resistance exercise [26] .

Our finding that standing core exercise elicits significant circulatory and hemodynamic changes provides preliminary scientific evidence of its clinical use in rehabilitation. Indeed, early mobilization and ambulation have been advocated in rehabilitation [27]. Such core exercise requires relatively lower metabolic demand, which may be suitable for patients or athletes with lower functional capacity who attempt to re-establish trunk stability and mobility. However, some limitations warrant caution when interpreting the results of our study. First, we only included young male healthy and active participants in the study, limiting our findings generalized to other populations who might also benefit from core muscle exercise [7, 10]. Second, there is no proper or accepted way to equate KE and core exercise conditions due to the natural difference between exercise modes (resistance exercise vs. endurance exercise). Participants were instructed to exercise to exhaustion in the active control session (KE) to maximize the responses.

In conclusion, standing core exercise elicits circulatory and hemodynamic changes only when the rotational cadence is set at a faster cadence; the acute responses elicited by slower cadence are less pronounced, which should be considered when including this type of exercise into program.

## Data Availability

The datasets used and/or analyzed during the current study are available for the corresponding author on reasonable request.

## References

[1] Seals DR, Desouza CA, Donato AJ, Tanaka H (2008). Habitual exercise and arterial aging. J Appl Physiol.

[2] Anton MM, Cortez-Cooper MY, DeVan AE, Neidre DB, Cook JN, Tanaka H (2006). Resistance training increases basal limb blood flow and vascular conductance in aging humans. J Appl Physiol.

[3] Miyachi M, Tanaka H, Yamamoto K, Yoshioka A, Takahashi K, Onodera S (2001). Effects of one-legged endurance training on femoral arterial and venous size in healthy humans. J Appl Physiol..

[4] Pedlar CR, Brown MG, Shave RE, Otto JM, Drane A, Michaud-Finch J (2018). Cardiovascular response to prescribed detraining among recreational athletes. J Appl Physiol.

[5] Sugawara J, Hayashi K, Kaneko F, Yamada H, Kizuka T, Tanaka H (2004). Reductions in basal limb blood flow and lumen diameter after short-term leg casting. Med Sci Sports Exerc.

[6] Tarnanen SP, Siekkinen KM, Hakkinen AH, Malkia EA, Kautiainen HJ, Ylinen JJ (2012). Core muscle activation during dynamic upper limb exercises in women. J Strength Cond Res.

[7] Sasaki S, Tsuda E, Yamamoto Y, Maeda S, Kimura Y, Fujita Y (2019). Core-muscle training and neuromuscular control of the lower limb and trunk. J Athl Train.

[8] Granacher U, Gollhofer A, Hortobagyi T, Kressig RW, Muehlbauer T (2013). The importance of trunk muscle strength for balance, functional performance, and fall prevention in seniors: a systematic review. Sports Med.

[9] Granacher U, Lacroix A, Muehlbauer T, Roettger K, Gollhofer A (2013). Effects of core instability strength training on trunk muscle strength, spinal mobility, dynamic balance and functional mobility in older adults. Gerontology.

[10] Haruyama K, Kawakami M, Otsuka T (2017). Effect of core stability training on trunk function, standing balance, and mobility in stroke patients. Neurorehabil Neural Repair.

[11] Lin H-F, Wang S-C, Cheng H-M, Sugawara J. Homebased standing core exercise training improves femoral blood flow but not arterial stiffness in middle-aged to older adults. Artery Res. 2021 (**In Press**).

[12] Saeterbakken AH, Fimland MS (2012). Muscle activity of the core during bilateral, unilateral, seated and standing resistance exercise. Eur J Appl Physiol.

[13] Sung PS, Lee KJ, Park WH (2012). Coordination of trunk and pelvis in young and elderly individuals during axial trunk rotation. Gait Posture.

[14] Hughes WE, Ueda K, Treichler DP, Casey DP (2015). Rapid onset vasodilation with single muscle contractions in the leg: influence of age. Physiol Rep..

[15] American College of Sports Medicine (2006). ACSM’s guidelines for exercise testing and prescription.

[16] Shoemaker JK, Hodge L, Hughson RL (1994). Cardiorespiratory kinetics and femoral artery blood velocity during dynamic knee extension exercise. J Appl Physiol.

[17] Sherwood A, Allen MT, Fahrenberg J, Kelsey RM, Lovallo WR, van Doornen LJ (1990). Methodological guidelines for impedance cardiography. Psychophysiology.

[18] Donato AJ, Uberoi A, Wray DW, Nishiyama S, Lawrenson L, Richardson RS (2006). Differential effects of aging on limb blood flow in humans. Am J Physiol Heart Circ Physiol.

[19] Dhindsa M, Barns J, DeVan AE, Nualnim N, Tanaka H (2008). Innovative exercise device that simulates horseback riding: cardiovascular and metabolic responses. Comp Exerc Physiol.

[20] Studinger P, Lenard Z, Kovats Z, Kocsis L, Kollai M (2003). Static and dynamic changes in carotid artery diameter in humans during and after strenuous exercise. J Physiol.

[21] Compton RO, Ulcak M, Gonzales JU (2015). The acute effect of fast and slow stepping cadence on regional vascular function. Int J Sports Med.

[22] Joyner MJ, Casey DP (2015). Regulation of increased blood flow (hyperemia) to muscles during exercise: a hierarchy of competing physiological needs. Physiol Rev.

[23] Tanaka H, Shimizu S, Ohmori F, Muraoka Y, Kumagai M, Yoshizawa M (2006). Increases in blood flow and shear stress to nonworking limbs during incremental exercise. Med Sci Sports Exerc.

[24] Segal SS, Jacobs TL (2001). Role for endothelial cell conduction in ascending vasodilatation and exercise hyperaemia in hamster skeletal muscle. J Physiol.

[25] McKay WP, Chilibeck PD, Daku BL (2007). Resting mechanomyography before and after resistance exercise. Eur J Appl Physiol.

[26] Thornton MK, Potteiger JA (2002). Effects of resistance exercise bouts of different intensities but equal work on EPOC. Med Sci Sports Exerc.

[27] Kottke TE, Caspersen CJ, Hill CS (1984). Exercise in the management and rehabilitation of selected chronic diseases. Prev Med.

